# Supplementary material for: Determinants of Post-COVID-19 Conditions among SARS-CoV-2-Infected Patients in Saudi Arabia: A Web-Based Cross-Sectional Study
Source: Diseases. 2022 Aug 23;10(3):55. doi: 10.3390/diseases10030055 (PMC9497712; doi:10.3390/diseases10030055)
Supplement: Supplementary file 1 [file diseases-10-00055-s001.zip › diseases-1860286-Supplementary.pdf]

Questionnaire

Demographic data:

- **Gender**
  - Male
  - Female

- **Age**
  - < 25
  - 25–40
  - 40-60
  - 60 or more

- **Body mass index**
  - underweight
  - Normal
  - Overweight
  - obese

- **Smoking status**
  - Smoking
  - Nonsmoker
  - Current smoker
  - Ex-smoker

- **Alcohol habit**
  - Yes
  - No

- **Number of comorbid disorders**
  - 0
  - 1
  - 2
  - 3
  - 4 or more

- **Type of comorbid disease you have (multiple answers)**
  - none
  - Diabetes
  - Hypertension
  - Obesity
  - Chronic respiratory disease
  - Cardiovascular disease

- Liver disease
- Psychiatric disorders
- Renal impairment
- Other

- **Under chronic medication**
  - Yes
  - No

Acute phase of covid19 infection

- **Acute Covid19 severity**
  - Asymptomatic
  - Mild
  - Moderate
  - Severe
  - Critical

- **Symptoms at onset**
  - 0
  - 1
  - 2
  - 3
  - 4
  - 5 or more

- **Hospital admission during illness**
  - Yes
  - No

- **Length of in-hospital day**
  - Open answer

- **History of ICU admittance**
  - yes
  - no

- **Length of in-ICU Day**
  - Open answer

- **Need of oxygen therapy**
  - Yes
  - No

- **Treatment taken during the acute attack were recorded**

- vitamins
- minerals
- corticosteroid
- analgesics
- antipyretic

Post-covid symptoms

- **The number of complications**
  - 0
  - 1
  - 2
  - 3
  - 4
  - 5 or more

- **The type of complication**
  - Fatigue
  - Anosmia\dysgeusia
  - Neurological disorders
  - Rheumatological disorders
  - Dyspnea
  - Psychiatric disorders
  - Hair loss
  - Cutaneous lesion
  - Gastrointestinal disorders
  - Chest pain
  - Ocular symptoms
  - Other

- **The mean duration since the onset of the symptoms**
  - (Open question)
